# Rheumatism and Gout

**Published:** 1904-03-12

**Authors:** 


### Rheumatism and Gout.

Poynton and W. V. Shaw[1] oppose the view that rheumatism depends on indefinite sepsis. Thus the staphylococcus aureus, a common cause of pyæmia, is easily distinguished from the diplococcus rheumaticus. It was not found in 25 fatal cases of rheumatism, and when injected into animals caused multiple abscesses, while the diplococcus under precisely similar methods produced the lesions of rheumatism. When the two infections were injected together symptoms of pyæmia and of rheumatism appeared together, but distinct. The question of the infectivity of acute rheumatism has not often been brought forward, but occasionally outbreaks of several cases in one house at the same time have been observed. Thus Turner records a case where three persons were attacked, and E. Sydney Hawthorne[2] gives one where five persons were infected and one died. Each of them was seen by two medical men so that the diagnosis was not in doubt, and it did not appear that any local or climatic causes led to the coincidence of the five cases. Josué and Salomon claim[3] that marked cerebral changes are shown by Nissl's method of staining in acute rheumatism. The chromatophile granules disappear, many of the nerve cells are more or less destroyed by neurophages of two kinds, while diplococci may be found in some of the vessels of the brain and pia mater.

Epistaxis was pointed out as an early symptom of acute rheumatism in children some time ago by Sidney Phillips. H. G. Langwell has recently recorded two well-marked instances. One of the patients had epistaxis at the commencement of three attacks of acute rheumatism out of four from which he had suffered. The patients were brother and sister, and neither were subject to epistaxis at other times. The author remarks on the absence in most text-books of all reference to this symptom of rheumatism. *Aspirin* and *mesotan* continue to find favour as remedies. Both are derived from salicylic acid, and the latter being easily decomposed by water, must be mixed with oil, and is then applied to the skin, through which it is rapidly absorbed. It is recommended as a substitute for oil of gaultheria, the odour of which is very nauseous to some people, but it is doubtful whether it passes more rapidly through the skin than salicylic acid dissolved in lanolin. Posselt[4] used mesotan in 50 cases, and reports very favourably of it. He advises that it should be mixed with equal parts of olive oil and smeared on a different part of the skin each time to avoid irritation. No oil silk should be applied over it. Mackey[5] used aspirin in about the same number of cases, and considers it is free from most of the disadvantages of salicylates and pleasant to take. He points out that the percentage of heart lesions is far less under the alkali treatment than when reliance is placed on salicylates (1 in 54, compared with 1 in 12 according to the statistics of St. George's Hospital), and he therefore agrees with Ewart, Luff, and Burney Yeo in giving alkalies as well as salicylates. In the case of aspirin alkalies can only be given at a different hour, as they are incompatible with it. *Lumbago* is the subject of an interesting lecture by Gowers.[6] He considers the pain is due to compression of the "muscle spindles," which are certain bodies existing in the interstitial tissue between the muscles, and in which the afferent nerves terminate. The bodies are partly composed of fibrous tissue, and an inflammation of them spreads to the fibrous attachments of the muscles, and even to the fasciæ, and fibrous sheaths of the nerves, such as that of the sciatic. Hence he regards the process as a fibrositis. A similar affection is seen in brachial myalgia, where any slight passive movement causes a violent, painful, muscular contraction so severe that anchylosis of the joints is often supposed to exist. But if the attention is taken away, or the other muscles of the arm are fixed, gentle passive movement is possible. This affection may also spread to the nerve sheaths and produce true neuritis with tenderness of the nerves, electrical changes, and wasting of the muscles; or the affection may be limited perhaps to the insertion of a single muscle such as the deltoid. Besides the rheumatic forms of this fibrositis, there is a traumatic one such as we see in railway accidents, or strains involving the attachments of the muscles to the sacrum. A more or less painless form is seen in the contraction of the palmar fasciæ. These latter types are often extremely chronic, while lumbago, stiff neck, and the other rheumatic forms are less so. In the early stages rest, diaphoretics, lithia, and colchicum, superheated air, and, later on, massage and weak faradic currents are of value. Counter irritation and possibly daily deep injections of eucaine may give relief in obstinate cases. In reply to this lecture W. Bruce[7] argues that sciatica and brachial myalgia are due to deposits of urate of soda. A. M. Watts remarks on the invariable deposit of lithates in the urine in lumbago and the relief given by full doses of belladonna and potash, and Jacobi speaks of the indurations to be felt in the affected muscles under skilled palpation, as the Swedish writers teach, and the "doughy" feel of the muscles in acute cases. Edgeworth[8] describes two forms of lumbago, one in which the pain only occurs on movement and the other where it is constant but increased by movement and accompanied by tender spots outside the erector spinæ, and often by sciatic neuritis. He finds that salicylates in large doses (20 grains every three hours) or ammonium chloride in half drachm doses are of great value. In the milder type relief is given rapidly by a liniment containing one part of tincture of capsicum to five of water. Fagge praises the effects of potash and tincture of hyoscyamus.

**Sciatica.**—Fedderson[9] considers that in genuine sciatica, besides the typical pain of a more or less paroxysmal type, there is generally a row of tender spots. If it is of the neuritic variety, there will be patches of anæsthesia, loss of reflexes, muscular atrophy, a fall of the cutaneous temperature in the limb, and even paresis. Among little noticed causes are gonorrhœa, syphilis, malaria, tuberculosis. Diabetes, gout, and rheumatism, the latter with patches of myositis, are frequent causes; chlorosis an occasional one. He recommends rest in bed with the knee flexed on a pillow. After aperients he gives large doses of salicylates for a few days and then smaller ones, also iodide, methylene blue, or arsenic. Later on galvanism, hot douches, and movement treatment.

[1] Lancet, Jan. 9. [2] Brit. Med. Jour., Dec. 26. [3] Lancet, Nov. 21. [4] Clin. Excerpt, Jan. 1904. [5] Lancet, Nov. 7. [6] Brit. Med. Jour., Jan. 16. [7] Ibid., Jan. 23. [8] Ibid., Jan. 30. [9] Ibid., Jan. 16.

